# Development and validation of a predictive model for the risk of sarcopenia in the older adults in China

**DOI:** 10.1186/s40001-024-01873-w

**Published:** 2024-05-09

**Authors:** Qiugui Li, Hongtao Cheng, Wenjiao Cen, Tao Yang, Shengru Tao

**Affiliations:** 1 School of Nursing, Jinan University, Guangzhou, Guangdong China; 2 Department of Neurosurgery, The First Affiliated Hospital of Jinan University, Guangzhou, Guangdong China; 3 Department of Healthcare-Associated Infection Management, The First Affiliated Hospital of Jinan University, Guangzhou, Guangdong China

**Keywords:** Sarcopenia, Predictive model, CHARLS, Nomogram

## Abstract

**Background:**

Sarcopenia is a progressive age-related disease that can cause a range of adverse health outcomes in older adults, and older adults with severe sarcopenia are also at increased short-term mortality risk. The aim of this study was to construct and validate a risk prediction model for sarcopenia in Chinese older adults.

**Methods:**

This study used data from the 2015 China Health and Retirement Longitudinal Study (CHARLS), a high-quality micro-level data representative of households and individuals aged 45 years and older adults in China. The study analyzed 65 indicators, including sociodemographic indicators, health-related indicators, and biochemical indicators.

**Results:**

3454 older adults enrolled in the CHARLS database in 2015 were included in the final analysis. A total of 997 (28.8%) had phenotypes of sarcopenia. Multivariate logistic regression analysis showed that sex, Body Mass Index (BMI), Mean Systolic Blood Pressure (MSBP), Mean Diastolic Blood Pressure (MDBP) and pain were predictive factors for sarcopenia in older adults. These factors were used to construct a nomogram model, which showed good consistency and accuracy. The AUC value of the prediction model in the training set was 0.77 (95% CI = 0.75–0.79); the AUC value in the validation set was 0.76 (95% CI = 0.73–0.79). Hosmer–Lemeshow test values were *P* = 0.5041 and *P* = 0.2668 (both *P* > 0.05). Calibration curves showed significant agreement between the nomogram model and actual observations. ROC and DCA showed that the nomograms had good predictive properties.

**Conclusions:**

The constructed sarcopenia risk prediction model, incorporating factors such as sex, BMI, MSBP, MDBP, and pain, demonstrates promising predictive capabilities. This model offers valuable insights for clinical practitioners, aiding in early screening and targeted interventions for sarcopenia in Chinese older adults.

**Supplementary Information:**

The online version contains supplementary material available at 10.1186/s40001-024-01873-w.

## Background

With the rapid development of China’s economy, the country has gradually transitioned into an aging society. According to the findings of the seventh census conducted in 2020, China’s population aged 60 and above is projected to exceed 260 million, accounting for 18.7% of the total population. Within this demographic, those aged 65 and above are expected to surpass 190 million, making up 13.5% of the total population [1]. The increasing number of older adults will significantly escalate expenditures within the social security system, imposing a considerable financial burden on the government. Among the various health-related factors contributing to disability in older adults, sarcopenia and cognitive impairment have attracted significant academic and clinical attention [2]. Currently, China’s focus on sarcopenia has started relatively late, with general hospitals showing evident specialization and insufficient understanding of sarcopenia, which has yet to be categorized into a specific field.

Sarcopenia was traditionally defined by a reduction in muscle mass, but current research highlights the significance of muscle strength and its impact on physical function [3]. Since 2016, the World Health Organization has officially recognized sarcopenia as a disease and a pressing public health concern in aging populations [4–6]. The loss of skeletal muscle mass is central to sarcopenia and can lead to physical dysfunction [7]. Studies indicate that sarcopenia affects over a quarter of older adults in Chinese communities [8], with a global incidence among individuals over 60 ranging from 10 to 27% [9]. Various factors contribute to the onset of sarcopenia, including age, nutrition intake, physical inactivity, diseases, and iatrogenic factors [10]. Risk factors such as aging, malnutrition, smoking, and low BMI have been identified [11, 12], with higher prevalence observed in patients with chronic obstructive pulmonary disease [13], chronic heart failure [14] and chronic liver disease [15]. Furthermore, sarcopenia is associated with adverse outcomes like falls, functional decline, frailty, and mortality [16, 17].

Although there are a number of sarcopenia risk prediction models, they all have some limitations. For example, some models have small sample sizes, which may limit their generalizability and applicability to different older adults [18]. Additionally, some models rely on predictor variables that are difficult and time-consuming to collect, limiting their usefulness in real-world clinical applications [19]. Furthermore, there are models that do not capture all risk factors for sarcopenia, which may affect their predictive accuracy [20]. These limitations underscore the need for further research to develop more comprehensive and practical prediction models for sarcopenia.

In contrast to foreign studies primarily focused on disease-specific correlations with sarcopenia, research in our country is predominantly centered on current situations and influencing factors. Key factors such as age, exercise habits, number of diseases, malnutrition, risk of falls, and fatigue are identified as easily inducible factors for sarcopenia. This study aims to identify and incorporate these factors into the construction of a sarcopenia risk prediction model, providing valuable insights for early screening and intervention by clinical medical staff.

## Methods

### Data source

**Fig. 1:**
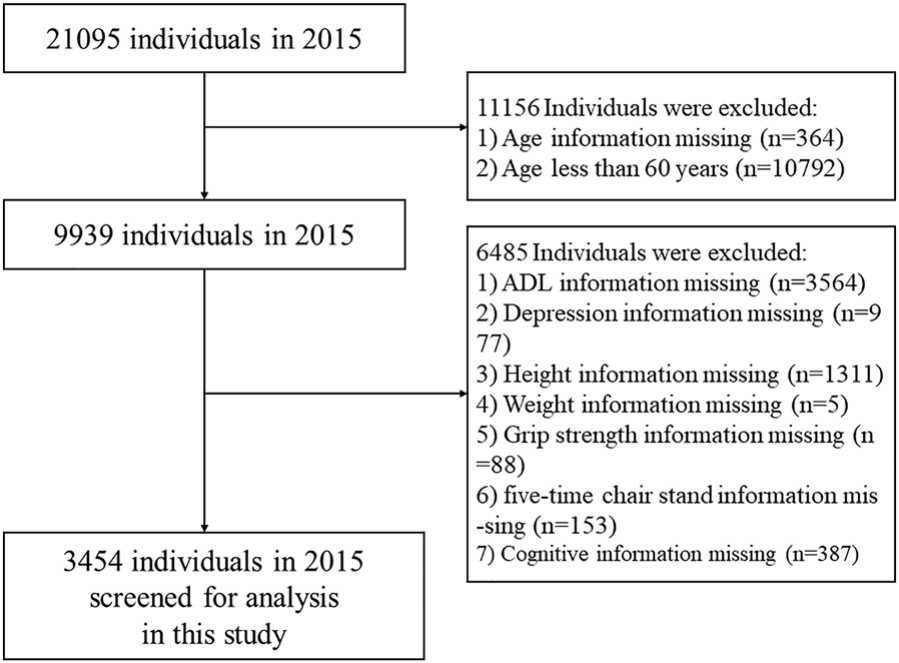
Flowchart of the study

We utilized data from the China Health and Retirement Longitudinal Study (CHARLS), publicly accessible at http://charls.pku.edu.cn. CHARLS is an ongoing longitudinal survey encompassing families and individuals aged 45 and older across 150 counties and 450 communities (villages) within 28 provinces, autonomous regions, and municipalities nationwide. Its comprehensive content spans demographic, economic, health, pension, and other pertinent information. Approval for this project was granted by the Biomedical Ethics Committee of Peking University (Beijing, China) (IRB00001052-11015), with our study adhering strictly to the principles outlined in the Declaration of Helsinki, and obtaining informed consent from all participants. Our analysis specifically focused on CHARLS2015 data, wherein after excluding subjects with missing data, a total of 3454 participants were ultimately included in our study cohort. Notably, our research targeted individuals aged 60 and above. The flowchart of the study is outlined in Fig. 1.

### Data extraction

#### Assessment of symptoms of sarcopenia

Sarcopenia was evaluated according to the criteria recommended by the AWGS2019 [7], which encompass muscle strength, physical performance and appendicular skeletal muscle mass (ASM). Handgrip strength (unit: kg) was assessed in both the dominant and non-dominant hand using the YuejianTM WL-1000 dynamometer. Participants were instructed to squeeze the handle as firmly as possible for 3 s. Measurements were taken twice for each hand, with a minimum interval of 15 s between trials. The recorded value represents the average of the maximum grip strength from both hands. The thresholds for low grip strength established by AWGS are < 28 kg for men and < 18 kg for women [21]. Physical performance decline, as per AWGS criteria, is defined as 5 times sitting time > 12 s or 6-m walking speed < 1 m/s [22]. ASM measurements were derived using validated anthropometric equations specifically developed for the Chinese population [23, 24]. The study demonstrated strong concordance between the ASM equation and Dual-Energy X-ray Absorptiometry (DXA) [23, 24]. In our study cohort, the cutoff for low muscle mass was determined based on sex-specific criteria, equating to a minimum of 20% of height-adjusted muscle mass (ASM/Ht^2^) [23–26]. Height and weight were recorded in centimeters and kilograms, respectively. Regarding sex, a value of 1 represents male and a value of 2 represents female. Consequently, individuals with ASM/Ht^2^ < 5.69 kg/m^2^ for females and ASM/Ht^2^ < 6.88 kg/m^2^ for males were classified as having low muscle mass. The ASM equation utilized is:

$$ASM\, = \,0.193*weight\left( {kg} \right)\, + \,0.107*height\left( {cm} \right) - 4.157*sex - 0.037*age\left( {years} \right) - 2.631$$

Sarcopenia manifests through a blend of diminished muscle strength, impaired physical performance, or decreased appendicular skeletal muscle mass. Diagnosis typically hinges on identifying low muscle strength, either alone or accompanied by reduced appendicular skeletal muscle mass. Individuals displaying low muscle strength, compromised physical performance, and diminished appendicular skeletal muscle mass were classified as having severe sarcopenia. For the purposes of this study, participants were segregated into two main groups: those with sarcopenia and those without.

#### Assessment of depressive symptoms

Depressive symptoms were assessed with a 10-item stream using the Center for Epidemiologic Studies Depression Scale (CES-D) to assess depressive mood and behavior. CESD pay attention to the individual's situation in the past week and rate it as “Rarely or none of the time (< 1 day)”, “Some or a little of the time (1-2 days)”, “Occasionally or a moderate amount of the time (3-4 days)”, and “Most or all of the time (5-7 days)” according to the frequency of symptoms, and assign 0, 1, 2, and 3 points, respectively, with higher scores representing individuals with more severe depressive symptoms. In this study, according to the research results of ROBERTS and his colleagues [27], CESD ≥ 16 is considered to have depressive symptoms, and < 16 is considered to have no depressive symptoms.

#### Assessment of cognitive function

CHARLS measures cognitive function in three parts: Telephone Interview for Cognitive Status (TICS), Word Recall, and Picture Drawing. The higher the score, the better the cognitive function. The TICS requires the subject to correctly name the year, month, day, day of the week, and season, and each correct answer is worth 1 point; the subject is required to correctly calculate 100-7, and each correct answer is worth 1 point, and the scores of the two parts are added together, the total score is 0–10 points, which mainly evaluates the subject’s orientation, calculation ability and attention. Word recall: the researchers read 10 words and asked the subjects to recall the 10 words in a short time and after answering several other questions, each correct recall of a word was recorded as 1 point, and the average score of the two words recalled was taken. A total score of 0–10 points was used to assess episodic memory ability. Picture drawing: the researcher provides a picture of two overlapping five-pointed stars and asks the subjects to draw the figure on a white piece of paper. If they can draw a similar figure, they get 1 point, and if they cannot, they get 0 points. It is used to evaluate the subject's visuospatial ability.

#### Assessment of activities of daily living

Activities of daily living include physical self-maintenance scale (PSMS) and instrumental activities of daily living (IADL). PSMS evaluates essential tasks like dressing, bathing, eating, getting out of bed, going to the toilet, controlling bowel and urine. Meanwhile, IADL assesses more complex activities such as shopping, cooking, doing housework, taking medicine, managing money and making phone calls. According to the degree, it is divided into “No, I don’t have any difficulty”, “I have difficulty but can still do it”, “Yes, I have difficulty and need help” and “I cannot do it”. These options correspond to scores of 1, 2, 3, and 4, respectively, indicating higher scores reflect greater impairment in the skill.

### Socio-demographic information

Socio-demographics include sex, age, marital status, education level, address and residence. Sex is defined as male and female. Education level was divided into no schooling, primary school, junior high school and above. Marital status was defined as married if the subject was currently married and living with a spouse; unmarried if the subject was currently separated, divorced, widowed, or never married. Address is divided into “Family house”, “Nursing home” and “Other”. Residence is divided into “The center of city/town”, “Combination zone between urban and rural areas”, “Village” and “Special area”.

### Health-related information

Within the health-related data examined as potential risk factors, a broad spectrum of conditions and indicators were included. These encompassed physical disabilities, neurological impairments such as brain damage, sensory deficits like blindness, deafness and muteness, as well as prevalent medical conditions including hypertension, dyslipidemia, diabetes, cancer and various chronic diseases affecting organs such as the lungs, liver, heart and kidneys. Mental health aspects such as emotional disturbances, memory-related ailments, and joint diseases or rheumatism were also considered. Other factors such as asthma, pain (specifically chronic pain), history of surgeries like cataract or hip fracture, usage of assistive devices like hearing aids, dental health indicators like tooth loss and lifestyle habits like smoking status, alcohol consumption, and social activity levels were evaluated. Additionally, variables related to accidents, falls, vision and hearing impairments and subjective health assessments were included. Specifically, aspects like distant vision, near vision, hearing ability and self-assessment of health status were categorized as “good”, “fair” or “poor”, while the remaining variables were dichotomized as “yes” or “no”. These variables can be directly obtained from the CHARLS questionnaire.

### Statistical methods

In this study, data from the CHARLS database in 2015 were selected for analysis. Continuous variables were expressed as medians and interquartile ranges, and rank sum tests were used to compare between groups; categorical variables were expressed as percentages, and *χ*^2^ tests or Fisher's exact tests were used to compare between groups. First, the data set is randomly divided into training set (*n* = 2417) and verification set (*n* = 1037) according to the ratio of 7:3. During this process, we set a random seed to ensure the randomization and repeatability of the sampling [28].

Utilizing a nomogram to depict the risk of sarcopenia among the older adults in China, we employed Least Absolute Selection and Shrinkage Operator (LASSO) regression analysis to construct and validate the model. We chose LASSO regression due to its capability to manage high-dimensional datasets with multicollinearity, effectively selecting variables and improving model interpretability. In contrast to Rigid and Elastic Net models, LASSO provides greater flexibility in variable selection and sparsity, making it the preferred choice for our specific research objectives and dataset characteristics. This choice ultimately leads to a more accurate and concise model. The primary R packages utilized in this study include: “mice”, “tableone”, “glmnet”, “rms”, “pROC” and “rmda”. First, LASSO regression analysis was performed on the training set data to select predictors of sarcopenia in Chinese older adults [29, 30]. Then, the tuning parameter (λ) suitable for LASSO regression analysis was determined by tenfold cross-validation, and the most significant features were screened using the LASSO algorithm. Finally, the selected predictors were included in the multivariate logistic regression analysis and the predictors with *P*-value < 0.05 were included in the nomogram model. The maximum missing value of all extracted variables does not exceed 20%, and multiple imputation is used to handle missing data [31].

In this study, the area under the receiver operating characteristic curve (AUROC) was used to determine the discriminative ability of the model. Calibration curves are used to determine the degree of agreement between predicted probabilities and observed results. Clinical validity was assessed by decision curve analysis (DCA). All data in this study were analyzed using R software (version 4.1.0). All tests were two-tailed and *P* < 0.05 was considered statistically significant.

## Results

### General information and clinical characteristics of the older adults

A total of 3454 older adult subjects (aged 60 years and older) were enrolled in this study, and the screening process for specific subjects is shown in Fig. 1. The general information and clinical characteristics of the subjects are listed in Table 1. There were 1708 men (49.4%) and 1746 women (50.6%). More detailed information is provided in a separate document (see supplement information).

**Table 1:** Baseline characteristics of the study population

**Variables**	Total (*n* = 3454)	Non-sarcopenia (*n* = 2457)	Sarcopenia (*n* = 997)	*P*-value
Sex (%)				< 0.001
Male	1708 (49.4)	914 (37.2)	794 (79.6)	
Female	1746 (50.6)	1543 (62.8)	203 (20.4)	
Marriage (%)				< 0.001
Married	2720 (78.7)	1895 (77.1)	825 (82.7)	
Unmarried	734 (21.3)	562 (22.9)	172 (17.3)	
Address (%)				0.976
Family housing	3420 (99.0)	2433 (99.0)	987 (99.0)	
Nursing home	4 (0.1)	3 (0.1)	1 (0.1)	
Other	30 (0.9)	21 (0.9)	9 (0.9)	
Residence (%)				0.160
The center of city/town	812 (23.5)	591 (24.1)	221 (22.2)	
Combination zone between urban and rural areas	246 (7.1)	161 (6.6)	85 (8.5)	
Village	2383 (69.0)	1695 (69.0)	688 (69.0)	
Special area	13 (0.4)	10 (0.4)	3 (0.3)	
Education (%)				< 0.001
Never went to school	1303 (37.7)	1020 (41.5)	283 (28.4)	
Primary school	1237 (35.8)	840 (34.2)	397 (39.8)	
Junior high school and above	914 (26.5)	597 (24.3)	317 (31.8)	
Physical disability (%)				0.693
No	3158 (91.4)	2243 (91.3)	915 (91.8)	
Yes	296 (8.6)	214 (8.7)	82 (8.2c)	
Brain damage (%)				0.789
No	3216 (93.1)	2290 (93.2)	926 (92.9)	
Yes	238 (6.9)	167 (6.8)	71 (7.1)	
Blind (%)				0.990
No	3030 (87.7)	2156 (87.7)	874 (87.7)	
Yes	424 (12.3)	301 (12.3)	123 (12.3)	
Deaf (%)				0.611
No	2877 (83.3)	2041 (83.1)	836 (83.9)	
Yes	577 (16.7)	416 (16.9)	161 (16.1)	
Dumb (%)				
No	3430 (99.3)	2442 (99.4)	988 (99.1)	0.477
Yes	24 (0.7)	15 (0.6)	9 (0.9)	
Hypertension (%)				0.746
No	2281 (66.0)	1618 (65.9)	663 (66.5)	
Yes	1173 (34.0)	839 (34.1)	334 (33.5)	
Dyslipidemia				0.394
No	2819 (81.6)	1996 (81.2)	823 (82.5)	
Yes	635 (18.4)	461 (18.8)	174 (17.5)	
Diabetes (%)				0.595
No	3052 (88.4)	2166 (88.2)	886 (88.9)	
Yes	402 (11.6)	291 (11.8)	111 (11.1)	
Cancer (%)				
No	3407 (98.6)	2424 (98.7)	983 (98.6)	1.000
Yes	47 (1.4)	33 (1.3)	14 (1.4)	
Chronic lung disease (%)				
No	2966 (85.9)	2125 (86.5)	841 (84.4)	
Yes	488 (14.1)	332 (13.5)	156 (15.6)	
Heart disease				0.378
No	2765 (80.1)	1957 (79.6)	808 (81.0)	
Yes	689 (19.9)	500 (20.4)	189 (19.0)	
Stroke (%)				
No	3371 (97.6)	2406 (97.9)	965 (96.8)	0.064
Yes	83 (2.4)	51 (2.1)	32 (3.2)	
Kidney disease (%)				0.017
No	3189 (92.3)	2251 (91.6)	938 (94.1)	
Yes	265 (7.7)	206 (8.4)	59 (5.9)	
Stomach disease (%)				0.899
No	2612 (75.6)	1860 (75.7)	752 (75.4)	
Yes	842 (24.4)	597 (24.3)	245 (24.6)	
Emotional and mental problems (%)				0.875
No	3402 (98.5)	2419 (98.5)	983 (98.6)	
Yes	52 (1.5)	38 (1.5)	14 (1.4)	
Diseases related to memory (%)				0.861
No	3426 (99.2)	2438 (99.2)	988 (99.1)	
Yes	28 (0.8)	19 (0.8)	9 (0.9)	
Joint disease or rheumatism (%)				0.290
No	2255 (65.3)	1618 (65.9)	637 (63.9)	
Yes	1199 (34.7)	839 (34.1)	360 (36.1)	
Asthma				0.531
No	3318 (96.1)	2364 (96.2)	954 (95.7)	
Yes	136 (3.9)	93 (3.8)	43 (4.3)	
Cataract surgery				0.279
No	3345 (96.8)	2385 (97.1)	960 (96.3)	
Yes	109 (3.2)	72 (2.9)	37 (3.7)	
Glaucoma				0.426
No	3357 (97.2)	2392 (97.4)	965 (96.8)	
Yes	97 (2.8)	65 (2.6)	32 (3.2)	
Hearing aid				0.918
No	3422 (99.1)	2435 (99.1)	987 (99.0)	
Yes	32 (0.9)	22 (0.9)	10 (1.0)	
Tooth loss				0.362
No	2987 (86.5)	2116 (86.1)	871 (87.4)	
Yes	467 (13.5)	341 (13.9)	126 (12.6)	
Pain				0.114
No	2240 (64.9)	1614 (65.7)	626 (62.8)	
Yes	1214 (35.1)	843 (34.3)	371 (37.2)	
Smoke				1.000
No	2380 (68.9)	1693 (68.9)	687 (68.9)	
Yes	1074 (31.1)	764 (31.1)	310 (31.1)	
Traffic accident (%)				0.855
No	3190 (92.4)	2271 (92.4)	919 (92.2)	
Yes	264 (7.6)	186 (7.6)	78 (7.8)	
History of falls (%)				0.414
No	2755 (79.8)	1969 (80.1)	786 (78.8)	
Yes	699 (20.2)	488 (19.9)	211 (21.2)	
Hip fracture (%)				1.000
No	3386 (98.0)	2409 (98.0)	977 (98.0)	
Yes	68 (2.0)	48 (2.0)	20 (2.0)	
Wearing glasses (%)				0.264
No	2274 (65.8)	1603 (65.2)	671 (67.3)	
Yes	1180 (34.2)	854 (34.8)	326 (32.7)	
Distant vision (%)				0.496
Good	867 (25.1)	615 (25.0)	252 (25.3)	
Fair	1855 (53.7)	1333 (54.3)	522 (52.4)	
Poor	732 (21.2)	509 (20.7)	223 (22.4)	
Near vision (%)				0.907
Good	911 (26.4)	645 (26.3)	266 (26.7)	
Fair	1801 (52.1)	1287 (52.4)	514 (51.6)	
Poor	742 (21.5)	525 (21.4)	217 (21.8)	
Hearing (%)				0.641
Good	997 (28.9)	720 (29.3)	277 (27.8)	
Fair	2001 (57.9)	1412 (57.5)	589 (59.1)	
Poor	456 (13.2)	325 (13.2)	131 (13.1)	
Social activity (%)				0.376
No	1466 (42.4)	1055 (42.9)	411 (41.2)	
Yes	1988 (57.6)	1402 (57.1)	586 (58.8)	
Drink (%)				0.073
No	2265 (65.6)	1588 (64.6)	677 (67.9)	
Yes	1189 (34.4)	869 (35.4)	320 (32.1)	
Self-assessment of health status (%)				0.401
Good	525 (15.2)	381 (15.5)	144 (14.4)	
Fair	1837 (53.2)	1289 (52.5)	548 (55.0)	
Poor	1092 (31.6)	787 (32.0)	305 (30.6)	
Depression				0.780
No	2928 (84.8)	2086 (84.9)	842 (84.5)	
Yes	526 (15.2)	371 (15.1)	155 (15.5)	
PSMS	6.00 (6.00, 6.00)	6.00 (6.00, 6.00)	6.00 (6.00,6.00)	0.305
IADL	6.00 (6.00,6.00)	6.00 (6.00, 6.00)	6.00 (6.00,6.00)	0.523
BMI	23.74 (21.45, 26.15)	23.92 (21.67, 26.32)	23.18 (20.85, 25.70)	< 0.001
Cognitive function score	11.50 (8.50, 14.00)	11.50 (8.50, 14.00)	11.50 (8.50, 14.00)	0.471
Night sleep duration (h)	6.00 (5.00, 8.00)	6.00 (5.00, 8.00)	6.00 (5.00, 8.00)	0.648
Lunch break time (min)	30.00 (0.00, 60.00)	30.00 (0.00, 60.00)	30.00 (0.00, 60.00)	0.468
Age (years)	67.00 (63.00, 73.00)	67.00 (63.00, 73.00)	67.00 (63.00, 73.00)	0.313
WBC (1000)	5.77 (4.80, 6.92)	5.73 (4.80, 6.95)	5.80 (4.84, 6.87)	0.816
HGB (g/dl)	13.70 (12.60, 14.80)	13.70 (12.60, 14.80)	13.70 (12.70, 14.80)	0.425
HCT (%)	41.40 (38.10, 44.80)	41.40 (38.10, 44.90)	41.40 (38.10, 44.80)	0.740
PLT (10^9^/L)	202.50 (161.00, 244.00)	203.00 (163.00, 244.00)	200.00 (155.00, 244.00)	0.128
MCV (fl)	91.85 (87.90, 95.90)	91.80 (88.00, 95.90)	91.90 (87.60, 95.80)	0.987
TG (mg/dl)	118.58 (84.96, 177.65)	118.58 (84.96, 178.76)	119.47 (83.19, 174.34)	0.643
CREA (mg/dl)	0.76 (0.65, 0.89)	0.76 (0.65, 0.89)	0.75 (0.65, 0.91)	0.330
BUN (mg/dl)	14.57 (12.32, 17.93)	14.57 (12.32, 17.93)	14.57 (12.32, 18.21)	0.506
HDL (mg/dl)	49.42 (42.47, 57.14)	49.42 (42.47, 56.76)	49.81 (42.86, 57.92)	0.038
LDL (mg/dl)	98.65 (80.69, 117.37)	98.46 (80.31, 117.37)	99.23 (81.47, 117.37)	0.733
CHO (mg/dl)	180.31 (158.01, 204.63)	179.92 (157.92, 204.25)	181.85 (158.69, 205.41)	0.562
GLU (mg/dl)	95.50 (88.29, 106.31)	95.50 (88.29, 108.11)	95.50 (88.29, 104.50)	0.141
CYSC (mg/l)	0.82 (0.71, 0.95)	0.82 (0.71, 0.95)	0.82 (0.72, 0.95)	0.465
UA (mg/dl)	4.80 (4.00, 5.70)	4.80 (3.90, 5.70)	4.80 (4.00, 5.70)	0.690
CRP (mg/l)	1.40 (0.80, 2.70)	1.40 (0.80, 2.70)	1.40 (0.70, 2.60)	0.328
HBALC (%)	5.80 (5.50, 6.10)	5.80 (5.50, 6.10)	5.80 (5.50, 6.10)	0.374
MSBP (mmHg)	125.33 (113.00, 140.33)	125.67 (113.33, 140.00)	125.00 (112.00, 142.33)	0.752
MDBP (mmHg)	74.67 (67.33, 82.67)	75.67 (67.67, 84.00)	73.00 (66.33, 80.00)	< 0.001

Medians and interquartile ranges (25th and 75th percentiles) were calculated for continuous variables and frequencies and percentages for categorical variables. The Wilcoxon rank sum test was used to compare group differences for continuous variables and Chi-squared tests for categorical variables

*PSMS* Physical Self-Maintenance Scale, *IADL* Instrumental Activities of Daily Living, *BMI* body mass index, *WBC* white blood cell, *HGB* hemoglobin, *HCT* hematocrit, *PLT* platelets, *MCV* mean corpuscular volume, *TG* triglycerides, *CREA* creatinine; *BUN* blood urea nitrogen; *HDL* high density lipoprotein cholesterol, *LDL* high density lipoprotein cholesterol, *CHO* total cholesterol, *GLU* glucose, *CYSC* cystatin C, *UA* uric acid, *CRP* C-reactive protein, *HBALC* glycated hemoglobin, *MSBP* mean systolic blood pressure, *MDBP* mean diastolic blood pressure

### Prevalence and associated variables of sarcopenia

The prevalence of sarcopenia was 28.8% (997/3454). There were significant differences in sex, BMI, and MDBP between the two groups of older adults (*P* < 0.05). According to clinical experience [32, 33], pain and MSBP were included in the model, and significant differences were found between the two groups of older adults. In the older adults, 2417 (70%) and 1037 (30%) were randomly assigned to the training and validation sets, respectively. The comparison of training and validation sets in the supplement information shows no significant difference between the two groups (*P* > 0.05).

### LASSO logistic regression

**Fig. 2:**
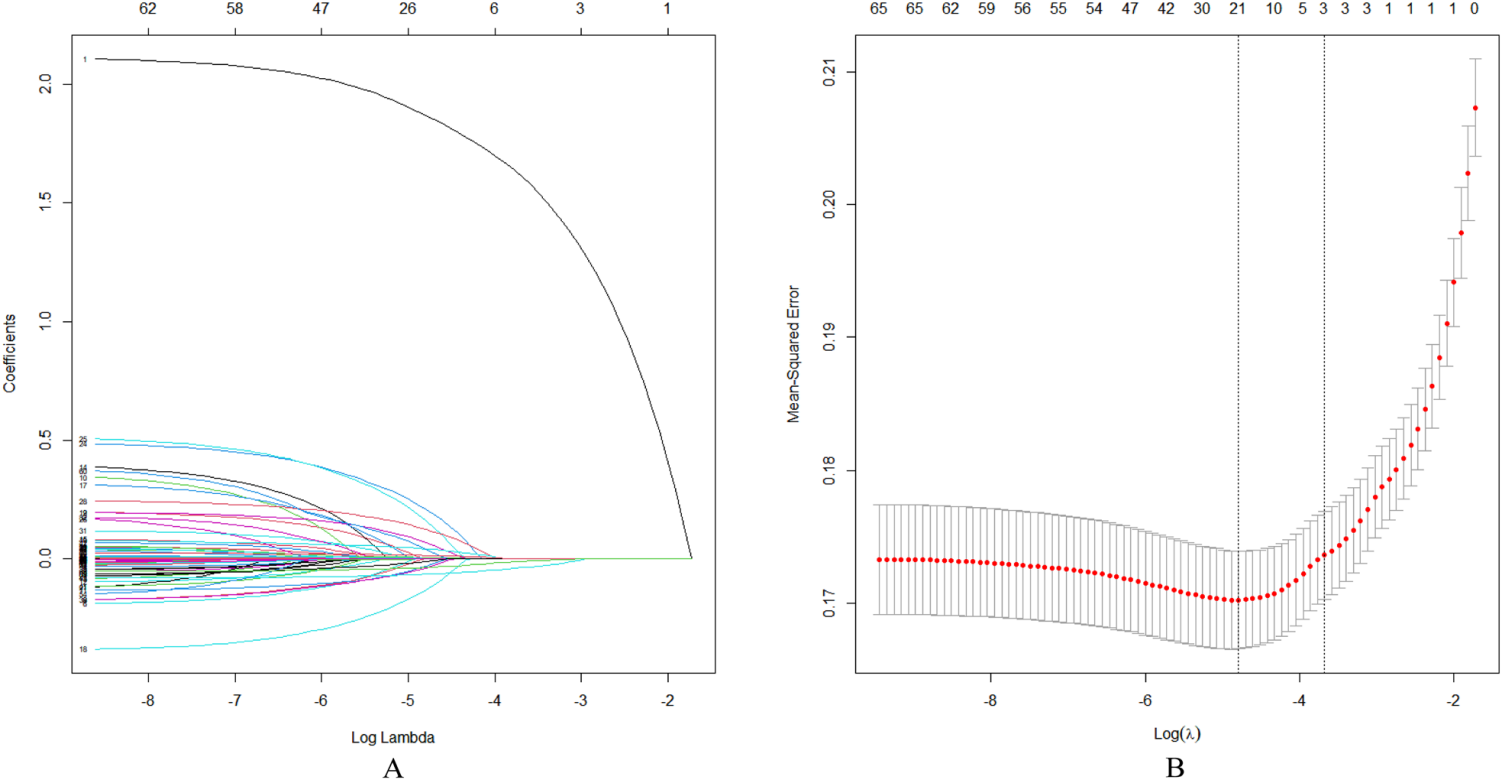
Demographic and clinical feature selection using the LASSO regression model. **A** According to the logarithmic (lambda) sequence, a coefficient profile was generated, and non-zero coefficients were produced by the optimal lambda. **B** The optimal parameter (lambda) in the LASSO model was selected via tenfold cross-validation using minimum criteria. The partial likelihood deviation (binomial deviation) curve relative to log (lambda) was plotted. A virtual vertical line at the optimal value was drawn using one SE of minimum criterion (the 1-SE criterion)

In this investigation, non-zero coefficients were identified as potential predictors of frailty through the LASSO regression model (Fig. 2A and Fig. 2B). Subsequently, these underlying factors linked with sarcopenia were incorporated into multiple logistic regression models utilizing the ‘rms’ package within the ‘R’ software environment. Ultimately, it was found that sex (*P* < 0.001), BMI (*P* < 0.001), MSBP (*P* < 0.001), MDBP (*P* < 0.001) and pain (*P* = 0.015) were correlated with sarcopenia in the older adults (Table 2).

**Table 2:** The prediction model with multivariate logistic regression

Variables	OR (95% CI)	*P*-value
Sex		< 0.001
Male	7.37 (5.95–9.17)	
Female	Reference	
BMI	0.92 (0.89–0.94)	< 0.001
MSBP	1.02 (1.01–1.03)	< 0.001
MDBP	0.94 (0.93–0.96)	< 0.001
Pain		0.015
No	Reference	
Yes	1.28 (1.04 -1.57)	

*OR* odds ratio, *CI* confidence interval, *BMI* body mass index, *MSBP* mean systolic blood pressure, *MDBP* mean diastolic blood pressure

### Developing predictive models

**Fig. 3:**
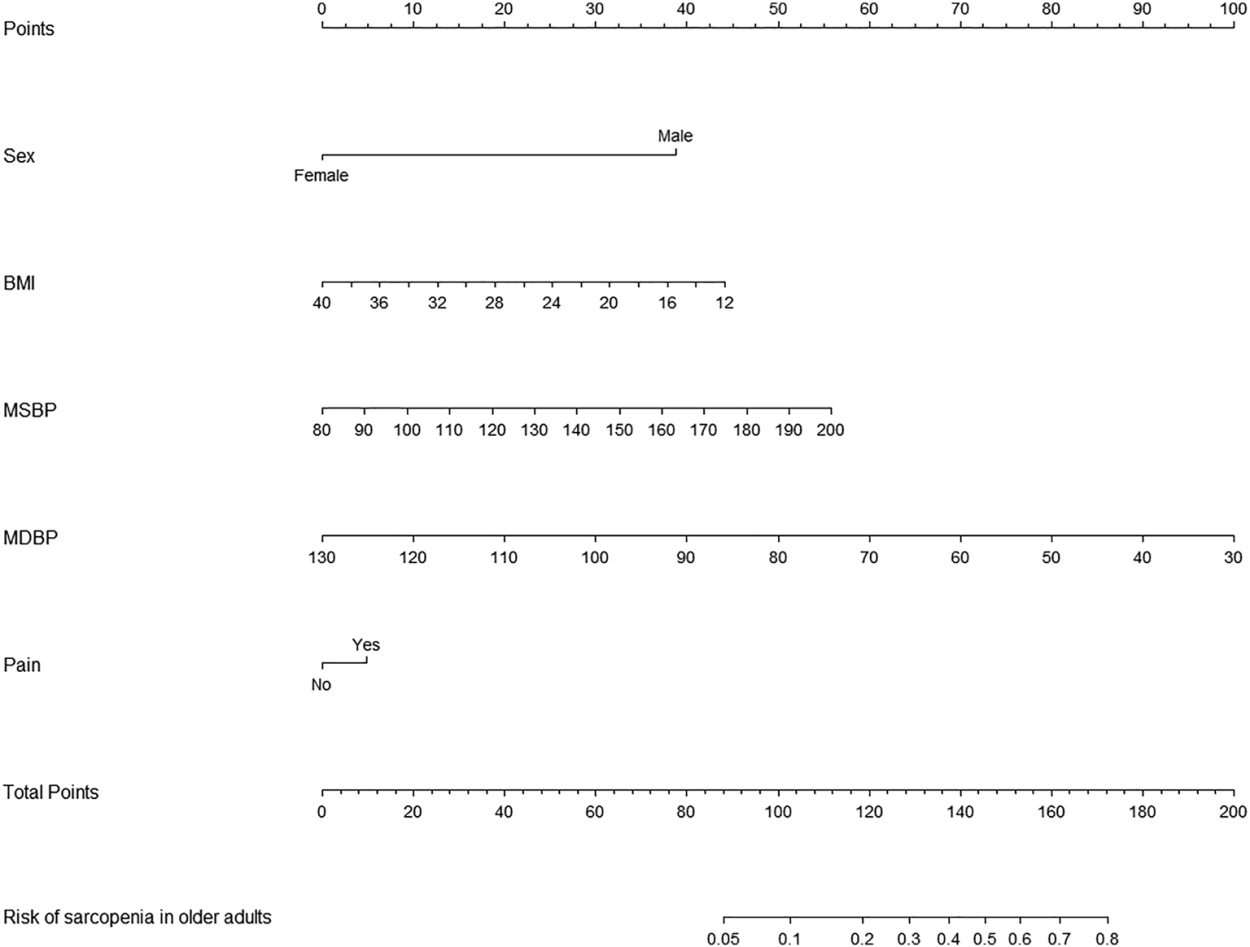
A nomogram for predicting sarcopenia in the older adults in China

Based on tenfold cross-validation, LASSO regression analysis was used to screen the best predictors of the model. Multiple logistic regression was used to build the prediction model. The variance inflation factor (VIF) test was performed, and the VIF values of all variables were < 4. Without covariance, the model fits well. The predictive model consists of variables with a *P*-value of less than 0.05 in a multivariate logistic regression. These variables included sex, BMI, MSBP, MDBP, pain as predictors. The prediction model adopts nomogram, which can be used to quantitatively predict the risk of sarcopenia in the older adults (Fig. 3).

### Validating predictive models

**Fig. 4:**
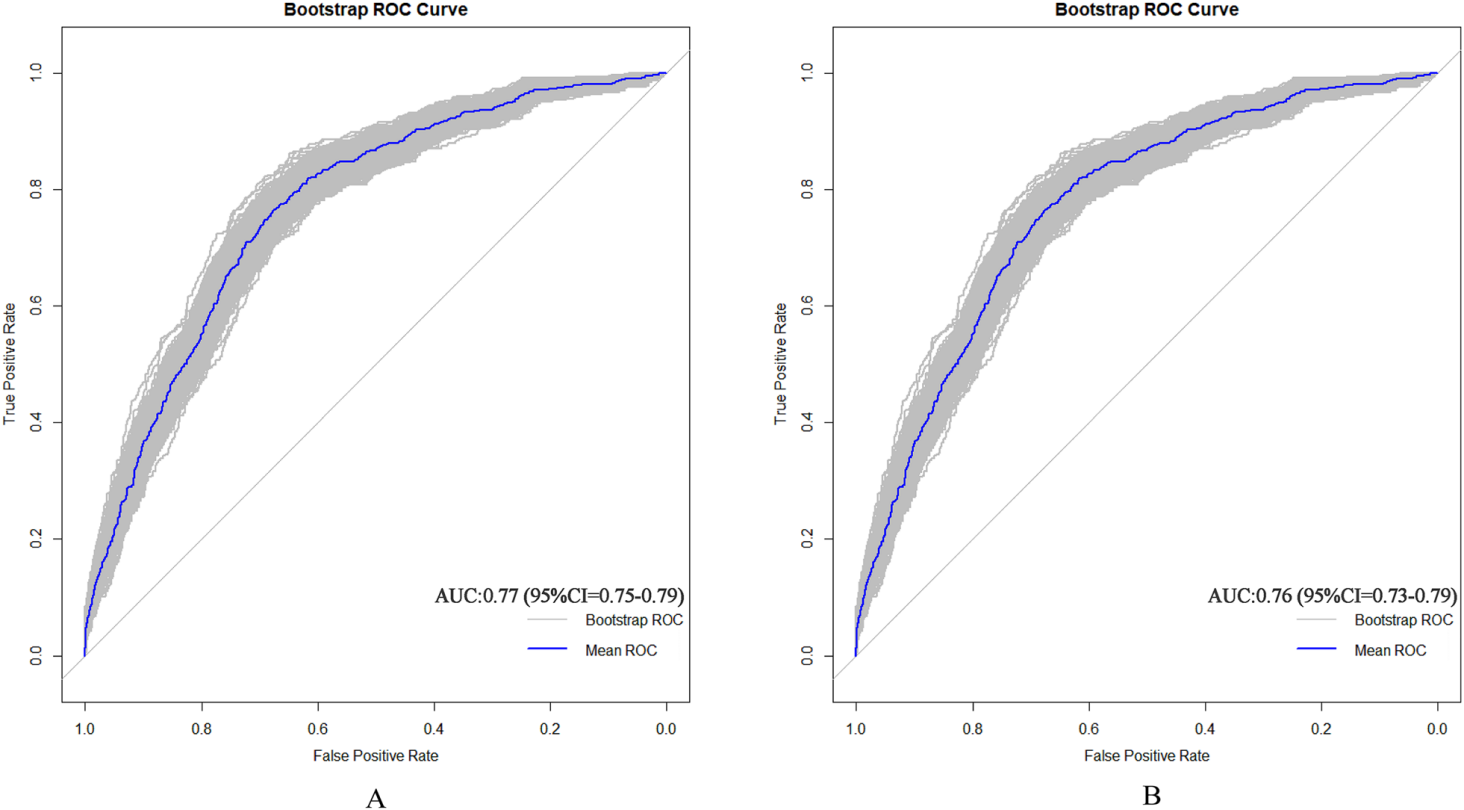
A Nomogram ROC curves generated from the training dataset. B Nomogram ROC curves generated using the validation dataset

AUC (area under curve) is a statistical metric that measures the performance of a classifier, specifically indicating the probability that a randomly chosen positive sample will rank higher than a randomly chosen negative sample. It is commonly utilized to assess the effectiveness of machine learning models. AUC values were computed to evaluate the discriminative power of the prediction model, by examining the incidence of sarcopenia among older adults in both the training and validation datasets. As shown in Fig. 4A, B, the AUC value of the predictive model in the training set was 0.77 (95% CI = 0.75–0.7901); the AUC value in the validation set was 0.76 (95% CI = 0.7287–0.7904). These data suggest that the nomogram has good discriminative power and predictive value, correctly identifying sarcopenic and non-sarcopenic patients.

### Correcting the predictive model

**Fig. 5:**
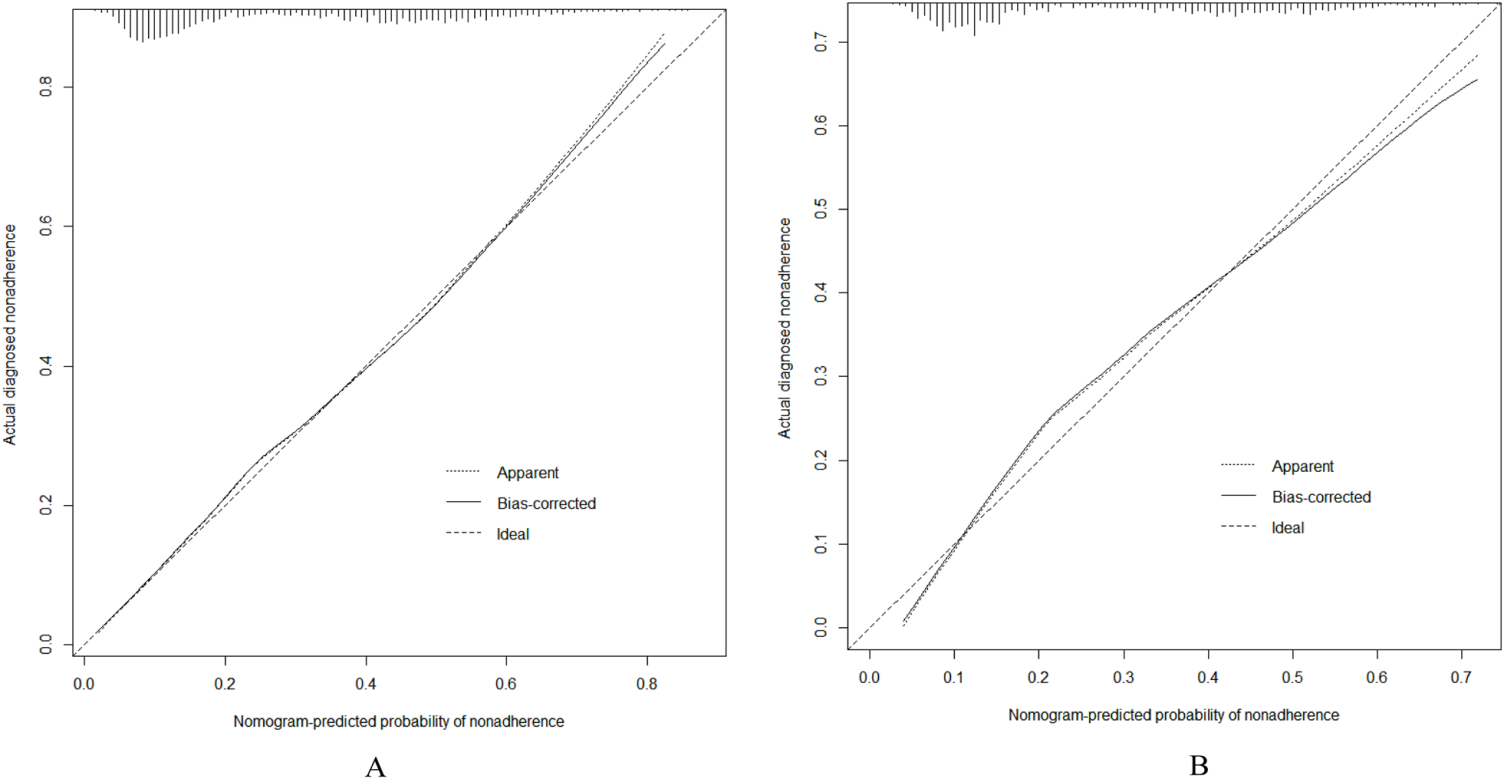
A Calibration plots for training dataset. B Calibration plots for validation dataset

Calibration plots and the Hosmer–Lemeshow goodness-of-fit test were used to evaluate the model plots (*P* > 0.05 indicated that the model fit was very good). The test results show that the model fits both the training set (*χ*^2^ = 7.305, *df* = 8, *p* = 0.5041) and the validation set (*χ*^2^ = 9.9748, *df* = 8, *p* = 0.2668) well. The calibration plots of the training and validation sets based on the multivariate logistic regression model are shown in Fig. 5A, B. The calibration curves of the modality maps showed that the predicted probability of sarcopenia for the training set (Fig. 5A) and the validation set (Fig. 5B) were highly consistent with the actual probability of sarcopenia.

### Clinical validity assessment

**Fig. 6:**
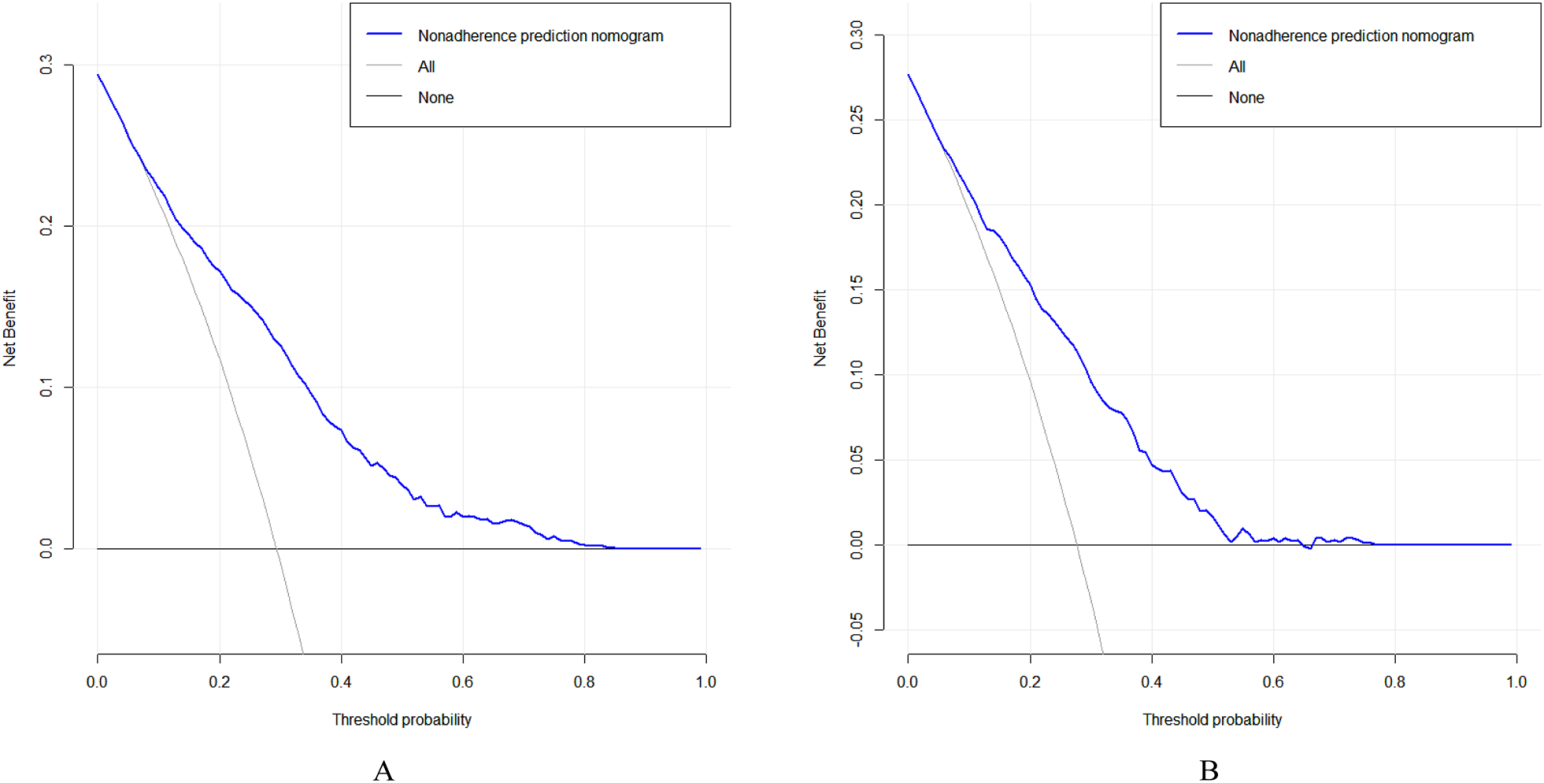
A DCA curves for training data set. B DCA curves for validation data set

The DCA method was used to evaluate the clinical validity of the model, and the results are shown in Figs. 6A, B. From the decision curve, the net benefit of the prediction model on the internal validation set is significantly higher than that of the two extreme cases, indicating that the nomogram model has better net benefit and prediction accuracy.

## Discussion

This study reveals that the prevalence of sarcopenia among the older adults in China stands at 28.8%, aligning with the findings reported by Cruz-Jentoft and his colleagues [34], which ranged from 1 to 29%. Sarcopenia can lead to reduced mobility, increased disability, falls, and risk of death [35, 36]. Therefore, identification of high-risk individuals is critical to preventing sarcopenia and its associated adverse outcomes.

This study shows that sex is a predictor of sarcopenia, and the results show that sarcopenia is related to sex, and the male population suffers from sarcopenia more often, which is consistent with previous research findings [37, 38]. The reason for the analysis may be that sarcopenia is caused by genetic inheritance or gene mutation. Since the disease-causing gene is located on the sex chromosome, and men have only one chromosome, one gene mutation is enough to cause the disease; women have two chromosomes, so two copies of muscular dystrophy can only be caused by a mutation in the gene. It is rare for a woman to inherit two disease-causing genes on her chromosomes, so men are more likely to have muscular dystrophy than women [39].Whether it is genetic or a mutation, there is currently no way to change the gene. The only way to control it is to delay development, improve symptoms, increase muscle strength, and prolong life through medication.

Our study revealed that BMI serves as a predictor of sarcopenia, with a lower BMI indicating a higher risk of sarcopenia, consistent with findings reported by Wu LC and colleagues [40]. This suggests a potential association between higher BMI and improved prognosis among older adults. However, it is important to note that while higher BMI may confer certain benefits, such as reduced risk of sarcopenia, it can also contribute to metabolic syndrome, posing physiological challenges for older individuals. Moreover, metabolic disorders associated with obesity may exacerbate malnutrition, perpetuating a detrimental cycle. A meta-analysis encompassing 26 studies [41] underscored the significant impact of different training modalities on muscle strength and physical performance in older adults with sarcopenia. Similarly, a systematic review [42] highlighted the positive effects of appropriate physical activity in enhancing muscle strength and flexibility, averting muscle atrophy and degeneration, and promoting blood circulation and metabolism, thereby fostering overall health in older adults aged 60 years and above [43]. Furthermore, the role of supplements in enhancing muscle mass and preventing metabolic syndrome onset is noteworthy. Selenium and magnesium, investigated in randomized controlled trials and dietary observational studies [44, 45], have shown potential associations with improved physical activity and muscle performance in older adults. Additionally, randomized controlled trials [46] have consistently demonstrated the efficacy of omega-3 fatty acids in preserving muscle mass and mitigating age-related muscle loss. In addressing the role of supplements, it is pertinent to mention Beta-hydroxy-beta-methylbutyrate (HMB), a metabolite of the essential amino acid leucine, which has garnered attention for its potential benefits in muscle health. Several studies have explored the effects of HMB supplementation on muscle mass preservation and physical function in older adults [47, 48]. These findings suggest that HMB may serve as a valuable adjunct to nutritional interventions and muscle training in mitigating the risk of sarcopenia among older adults. Therefore, early nutritional intervention and muscle training should be offered to older adults at risk for sarcopenia to reduce the risk of sarcopenia.

At the same time, this study also found that blood pressure is closely related to the occurrence of sarcopenia. High systolic blood pressure may reflect the stiffness of the blood vessels, which may reduce the ability of blood to flow to the muscles, resulting in an inadequate supply of nutrients to the muscles, thus increasing muscle loss. Low diastolic blood pressure may indicate that the heart is not pumping enough blood to the body during diastole, which may also affect the supply of nutrients to the muscles [49]. Both high systolic blood pressure and low diastolic blood pressure can be signs of physical decline in the older adults, and physical decline is closely related to sarcopenia. Therefore, in the future, in addition to paying attention to heart, brain and kidney complications, hypertensive patients should also pay attention to muscle loss.

In addition, this study shows that chronic pain, particularly chronic low back pain, is also associated with sarcopenia, which has a critical impact on spinal health because maintaining spinal function requires the involvement of strong lower back muscles. On the one hand, the decrease in muscle quantity and quality reduces muscle tolerance to exercise and makes muscles more susceptible to fatigue, which reduces their ability to maintain overall spinal stability. Spinal instability greatly increases the incidence of chronic low back pain. On the other hand, the decline in the function of the trunk muscles, especially the dorsi extensors, leads to a weakening of the muscles' suspension force on the spine, making it difficult for the body to maintain a normal upright posture, resulting in a severe forward tilt of the body. Leaning forward increases the work of the posterior muscles, fatigues the muscle tissue, and makes it impossible to keep the body upright, creating a vicious cycle that affects the patient's quality of life [50]. Therefore, the prevention and treatment of sarcopenia is a very important and urgent issue for spinal health in older adults.

The nomogram, constructed through multifactorial regression analysis, amalgamates various predictive indicators to represent the relationships between variables in the predictive model using scaled line segments on a common plane according to a predetermined ratio. It serves as a tool to forecast the probability of a clinical outcome event by summing the scores assigned to each predictor to derive a total score. Widely employed across diverse clinical domains, the nomogram stands as a common predictive model utilized in research endeavors. To further bolster the credibility of our findings, we recognize the importance of engaging with previous studies that have developed and validated nomograms, as this interaction could enhance the robustness of our research outcomes. In this study, we identified sex, BMI, MSBP, MDBP and pain as the main factors predicting sarcopenia in Chinese older adults. Our prediction model, constructed based on these five factors influencing sarcopenia development, exhibited good discrimination, calibration, and clinical validity. This suggests that the prediction model holds value for effectively identifying high-risk older adults with sarcopenia. The nomogram specifically quantifies the hazard ratio in terms of scores, allowing for the calculation of the probability of a certain outcome through simple calculations. It provides individualized risk assessment for each person, enhancing relevance and accuracy.

Therefore, the establishment of a prediction model for sarcopenia in older adults constitutes a novel achievement of this study. Nomograms, as efficient and accurate assessment tools, can assist clinical medical staff in objectively screening older adults at risk of sarcopenia, thereby providing a theoretical basis and starting point for formulating early prevention and intervention measures. Their clinical applicability is robust, aiding in the identification of patients at high risk for sarcopenia, enabling the implementation of early intervention plans, and facilitating individual health management in older adults.

This study has several limitations. Firstly, the absence of age-specific analysis based on different age groups is a notable gap. Sarcopenia, which involves a decrease in muscle mass due to age-related hormonal changes, would have benefited from a more granular examination across age brackets. Secondly, the CHARLS database lacked some potential predictors, such as dietary habits and nutritional status, limiting the scope of our analysis. Thirdly, the nomogram developed in this study is specific to data from China, and its generalizability to other regions and countries remains to be determined through external validation. Additionally, while pain was identified as an important factor in sarcopenia, the study did not delve into the specifics of pain, such as its location, intensity, and duration, which could have provided deeper insights. Furthermore, patients with impaired cognitive function were not excluded, and in some cases, family members assisted with self-reporting, potentially introducing biases into the results. Given these limitations, future research should aim to conduct prospective studies, incorporate more comprehensive predictor variables, and externally validate the model to enhance its generalizability and accuracy.

## Conclusions

Our sarcopenia risk prediction model based on CHARLS data provides a reliable and accurate tool for Chinese older adults. This model can help clinicians to identify high-risk patients earlier and take timely preventive and interventional measures to improve the quality of life and health outcomes of the older adults.

### Supplementary Information

Supplementary Material 1. Comparison between variables in the training and validation datasets

## Data Availability

The datasets generated during and/or analyzed during the current study are available in the CHARLS repository, http://charls.pku.edu.cn.
