# Supplementary material for: Development and validation of a predictive model for the risk of sarcopenia in the older adults in China
Source: Eur J Med Res. 2024 May 9;29:278. doi: 10.1186/s40001-024-01873-w (PMC11084063; doi:10.1186/s40001-024-01873-w)
Supplement: Supplementary file 1 — Supplementary Material 1. Comparison between variables in the training and validation datasets [file 40001_2024_1873_MOESM1_ESM.docx]

**Supplement information**

Comparison between variables in the training and validation datasets

| **Variables** | Total (n=3454) | Training set(n=2417) | Validation set(n=1037) | P-value |
| --- | --- | --- | --- | --- |
| Sarcopenia(%) |  |  |  | 0.332 |
| No | 2457(71.1) | 1707(70.6) | 750(72.3) |  |
| Yes | 997(28.9) | 710(29.4) | 287(27.7) |  |
| Sex(%) |  |  |  | 0.288 |
| Male | 1708(49.4) | 1210(50.1) | 498(48.0) |  |
| Female | 1746(50.6) | 1207(49.9) | 539(52.0) |  |
| Marriage(%) |  |  |  | 0.991 |
| Married | 2720(78.7) | 1904(78.8) | 816(78.7) |  |
| Unmarried | 734(21.3) | 513(21.2) | 221(21.3) |  |
| Address(%) |  |  |  | 0.392 |
| Family housing | 3420 (99.0) | 2393 (99.0) | 1027 (99.0) |  |
| Nursing home | 4 (0.1) | 4 (0.2) | 0 (0.0) |  |
| Other | 30 (0.9) | 20 (0.8) | 10 (1.0) |  |
| Residence(%) |  |  |  | 0.869 |
| The center of city/town | 812 (23.5) | 575 (23.8) | 237 (22.9) |  |
| Combination zone between urban and rural areas | 246 (7.1) | 170 (7.0) | 76 (7.3) |  |
| Village | 2383 (69.0) | 1662 (68.8) | 721 (69.5) |  |
| Special area | 13 (0.4) | 10 (0.4) | 3 (0.3) |  |
| Education(%) |  |  |  | 0.393 |
| Never went to school | 1303 (37.7) | 896 (37.1) | 407 (39.2) |  |
| Primary school | 1237 (35.8) | 868 (35.9) | 369 (35.6) |  |
| Junior high school and above | 914 (26.5) | 653 (27.0) | 261 (25.2) |  |
| Physical disability (%) |  |  |  | 0.562 |
| No | 3158(91.4) | 2205(91.2) | 953(91.9) |  |
| Yes | 296(8.6) | 212(8.8) | 84(8.1) |  |
| Brain damage(%) |  |  |  | 0.562 |
| No | 3216(93.1) | 2246(92.9) | 970(93.5) |  |
| Yes | 238(6.9) | 171(7.1) | 67(6.5) |  |
| Blind(%) |  |  |  | 0.512 |
| No | 3030(87.7) | 2114(87.5) | 916(88.3) |  |
| Yes | 424(12.3) | 303(12.5) | 121(11.7) |  |
| Deaf(%) |  |  |  | 0.243 |
| No | 2877(83.3) | 2001(82.8) | 876(84.5) |  |
| Yes | 577(16.7) | 416(17.2) | 161(15.5) |  |
| Dumb(%) |  |  |  | 0.446 |
| No | 3430(99.3) | 2398(99.2) | 1032(99.5) |  |
| Yes | 24(0.7) | 19(0.8) | 5(0.5) |  |
| Hypertension(%) |  |  |  | 0.958 |
| No | 2281(66.0) | 1595(66.0) | 686(66.2) |  |
| Yes | 1173(34.0) | 822(34.0) | 351(33.8) |  |
| Dyslipidemia |  |  |  | 0.298 |
| No | 2819(81.6) | 1984(82.1) | 835(80.5) |  |
| Yes | 635(18.4) | 433(17.9) | 202(19.5) |  |
| Diabetes(%) |  |  |  | 0.834 |
| No | 3052(88.4) | 2138(88.5) | 914(88.1) |  |
| Yes | 402(11.6) | 279(11.5) | 123(11.9) |  |
| Cancer(%) |  |  |  | 0.901 |
| No | 3407(98.6) | 2385(98.7) | 1022(98.6) |  |
| Yes | 47(1.4) | 32(1.3) | 15(1.4) |  |
| Chronic lung disease(%) |  |  |  | 0.395 |
| No | 2966(85.9) | 2084(86.2) | 882(85.1) |  |
| Yes | 488(14.1) | 333(13.8) | 155(14.9) |  |
| Heart disease |  |  |  | 0.666 |
| No | 2765(80.1) | 1940(80.3) | 825(79.6) |  |
| Yes | 689(19.9) | 477(19.7) | 212(20.1) |  |
| Stroke(%) |  |  |  | 0.072 |
| No | 3371(97.6) | 2351(97.3) | 1020(98.4) |  |
| Yes | 83(2.4) | 66(2.7) | 17(1.6) |  |
| Kidney disease(%) |  |  |  | 0.774 |
| No | 3189(92.3) | 2229(92.2) | 960(92.6) |  |
| Yes | 265(7.7) | 188(7.8) | 77(7.4) |  |
| Stomach disease(%) |  |  |  | 0.355 |
| No | 2612(75.6) | 1839(76.1) | 773(74.5) |  |
| Yes | 842(24.4) | 578(23.9) | 264(25.5) |  |
| Emotional and mental problems(%) |  |  |  | 0.119 |
| No | 3402(98.5) | 2375(98.3) | 1027(99.0) |  |
| Yes | 52(1.5) | 42(1.7) | 10(1.0) |  |
| Diseases related to memory(%) |  |  |  | 0.707 |
| No | 3426(99.2) | 2396(99.1) | 1030(99.3) |  |
| Yes | 28(0.8) | 21(0.9) | 7(0.7) |  |
| Joint disease or rheumatism(%) |  |  |  | 0.199 |
| No | 2255(65.3) | 1561(64.6) | 694(66.9) |  |
| Yes | 1199(34.7) | 856(35.4) | 343(33.1) |  |
| Asthma |  |  |  | 0.75 |
| No | 3318(96.1) | 2324(96.2) | 994(95.9) |  |
| Yes | 136(3.9) | 93(3.8) | 43(4.1) |  |
| Cataract surgery |  |  |  | 0.706 |
| No | 3345(96.8) | 2343(96.9) | 1002(96.6) |  |
| Yes | 109(3.2) | 74(3.1) | 35(3.4) |  |
| Glaucoma |  |  |  | 0.715 |
| No | 3357(97.2) | 2347(97.1) | 1010(97.4) |  |
| Yes | 97(2.8) | 70(2.9) | 27(2.6) |  |
| Hearing aid |  |  |  | 0.463 |
| No | 3422(99.1) | 2397(99.2) | 1025(98.8) |  |
| Yes | 32(0.9) | 20(0.8) | 12(1.2) |  |
| Tooth loss |  |  |  | 0.495 |
| No | 2987(86.5) | 2097(86.8) | 890(85.8) |  |
| Yes | 467(13.5) | 320(13.2) | 147(14.2) |  |
| Pain |  |  |  | 0.485 |
| No | 2240(64.9) | 1558(64.5) | 682(65.8) |  |
| Yes | 1214(35.1) | 859(35.5) | 355(34.2) |  |
| Smoke |  |  |  | 0.26 |
| No | 2380(68.9) | 1680(69.5) | 700(67.5) |  |
| Yes | 1074(31.1) | 737(30.5) | 337(32.5) |  |
| Traffic Accident(%) |  |  |  | 0.221 |
| No | 3190(92.4) | 2223(92.0) | 967(93.2) |  |
| Yes | 264(7.6) | 194(8.0) | 70(6.8) |  |
| History of falls(%) |  |  |  | 0.425 |
| No | 2755(79.8) | 1937(80.1) | 818(78.9) |  |
| Yes | 699(20.2) | 480(19.9) | 219(21.1) |  |
| Hip fracture(%) |  |  |  | 0.772 |
| No | 3386(98.0) | 2371(98.1) | 1015(97.9) |  |
| Yes | 68(2.0) | 46(1.9) | 22(2.1) |  |
| Wearing glasses(%) |  |  |  | 0.651 |
| No | 2274(65.8) | 1585(65.6) | 689(66.4) |  |
| Yes | 1180(34.2) | 832(34.4) | 348(33.6) |  |
| Distant vision(%) |  |  |  | 0.619 |
| Good | 867 (25.1) | 604 (25.0) | 263 (25.4) |  |
| Fair | 1855 (53.7) | 1310 (54.2) | 545 (52.6) |  |
| Poor | 732 (21.2) | 503 (20.8) | 229 (22.1) |  |
| Near vision(%) |  |  |  | 0.97 |
| Good | 911 (26.4) | 639 (26.4) | 272 (26.2) |  |
| Fair | 1801 (52.1) | 1257 (52.0) | 544 (52.5) |  |
| Poor | 742 (21.5) | 521 (21.6) | 221 (21.3) |  |
| Hearing(%) |  |  |  | 0.716 |
| Good | 997 (28.9) | 699 (28.9) | 298 (28.7) |  |
| Fair | 2001 (57.9) | 1392 (57.6) | 609 (58.7) |  |
| Poor | 456 (13.2) | 326 (13.5) | 130 (12.5) |  |
| Social activity(%) |  |  |  | 0.192 |
| No | 1466(42.4) | 1008(41.7) | 458(44.2) |  |
| Yes | 1988(57.6) | 1409(58.3) | 579(55.8) |  |
| Drink(%) |  |  |  | 0.666 |
| No | 2265(65.6) | 1591(65.8) | 674(65.0) |  |
| Yes | 1189(34.4) | 826(34.2) | 363(35.0) |  |
| Self-assessment of health status (%) |  |  |  | 0.732 |
| Good | 525 (15.2) | 375 (15.5) | 150 (14.5) |  |
| Fair | 1837 (53.2) | 1280 (53.0) | 557 (53.7) |  |
| Poor | 1092 (31.6) | 762 (31.5) | 330 (31.8) |  |
| Depression |  |  |  | 0.575 |
| No | 2928(84.8) | 2043(84.5) | 885(85.3) |  |
| Yes | 526(15.2) | 374(15.5) | 152(14.7) |  |
| PSMS | 6.00(6.00,6.00) | 6.00 (6.00, 6.00) | 6.00 (6.00, 6.00) | 0.247 |
| IADL | 6.00(6.00,6.00) | 6.00 (6.00, 6.00) | 6.00 (6.00, 6.00) | 0.767 |
| BMI | 23.74(21.45,26.15) | 23.72(21.43,26.22) | 23.75(21.50,25.96) | 0.932 |
| Cognitive function score | 11.50(8.50,14.00) | 11.50(8.50,14.00) | 11.50(8.50,14.00) | 0.389 |
| Night sleep duration(h) | 6.00(5.00,8.00) | 6.00 (5.00, 8.00) | 6.00(5.00, 8.00) | 0.239 |
| Lunch break time(min) | 30.00(0.00,60.00) | 30.00(0.00,60.00) | 30.00 (0.00, 60.00) | 0.599 |
| Age(years) | 67.00(63.00,73.00) | 67.00(63.00,73.00) | 67.00(63.00,73.00) | 0.85 |
| WBC(1000) | 5.77 (4.80, 6.92) | 5.80 (4.80, 6.90) | 5.70 (4.70, 6.96) | 0.513 |
| HGB(g/dl) | 13.70(12.60,14.80) | 13.60(12.60,14.80) | 13.80(12.70,15.00) | 0.055 |
| HCT(%) | 41.40(38.10,44.80) | 41.30(38.00,44.80) | 41.80(38.50,45.00) | 0.033 |
| PLT(109/L) | 202.50(161.00,  244.00) | 202.00(160.00,  245.00) | 203.00(162.00,  243.00) | 0.564 |
| MCV(fl) | 91.85(87.90,95.90) | 91.80(87.90,96.00) | 91.90(87.90,95.80) | 0.572 |
| TG(mg/dl) | 118.58(84.96,  177.65) | 119.47(84.96,  179.65) | 117.70(84.96,  170.80) | 0.431 |
| CREA(mg/dl) | 0.76 (0.65, 0.89) | 0.76 (0.65, 0.89) | 0.76 (0.65, 0.90) | 0.839 |
| BUN(mg/dl) | 14.57(12.32,17.93) | 14.57(12.32,17.93) | 14.57(12.04,17.93) | 0.623 |
| HDL(mg/dl) | 49.42(42.47,57.14) | 49.42(42.47,57.53) | 49.42(42.47,56.76) | 0.717 |
| LDL(mg/dl) | 98.65(80.69,  117.37) | 98.07(79.92,  116.22) | 100.77(82.24,  120.46) | 0.004 |
| CHO(mg/dl) | 180.31(158.01, 204.63) | 179.54(157.53, 203.86) | 182.24(159.46, 205.79) | 0.095 |
| GLU(mg/dl) | 95.50(88.29, 106.31) | 95.50(88.29, 106.31) | 95.50(88.29, 108.11) | 0.964 |
| CYSC(mg/l) | 0.82 (0.71, 0.95) | 0.82 (0.71, 0.95) | 0.82 (0.71, 0.95) | 0.443 |
| UA(mg/dl) | 4.80 (4.00, 5.70) | 4.80 (4.00, 5.70) | 4.80 (3.90, 5.70) | 0.529 |
| CRP(mg/l) | 1.40 (0.80, 2.70) | 1.40 (0.80, 2.70) | 1.40 (0.70, 2.60) | 0.263 |
| HBALC(%) | 5.80 (5.50, 6.10) | 5.80 (5.50, 6.10) | 5.80 (5.50, 6.20) | 0.564 |
| MSBP(mmHg) | 125.33(113.00, 140.33) | 125.67(113.00, 140.33) | 125.00(113.00, 140.67) | 0.805 |
| MDBP(mmHg) | 74.67(67.33, 82.67) | 74.67(67.33, 82.67) | 74.67(67.33, 83.33) | 0.702 |

Abbreviations: PSMS: Physical Self-Maintenance Scale; IADL: Instrumental Activities of Daily Living; BMI: Body Mass Index; WBC: White Blood Cell; HGB: Hemoglobin; HCT: Hematocrit; PLT : Platelets ; MCV: Mean Corpuscular Volume; TG: Triglycerides ; CREA : Creatinine; BUN : Blood Urea Nitrogen ; HDL : High Density Lipoprotein Cholesterol ;LDL: High Density Lipoprotein Cholesterol ; CHO : Total Cholesterol ; GLU : Glucose ;CYSC: Cystatin C ;UA: Uric Acid ;CRP: C-Reactive Protein ;HBALC: Glycated Hemoglobin ;MSBP: Mean Systolic Blood Pressure; MDBP: Mean Diastolic Blood Pressure.

Note: Medians and interquartile ranges (25th and 75th percentiles) were calculated for continuous variables and frequencies and percentages for categorical variables. The Wilcoxon rank sum test was used to compare group differences for continuous variables and Chi-squared tests for categorical variables.

Data description: The supplement information groups the participants according to the training set and validation set, and performs a statistical analysis of the basic characteristics of the two groups, with the results presented in tabular form.
